# Genome analyses of four *Wolbachia* strains and associated mitochondria of *Rhagoletis cerasi* expose cumulative modularity of cytoplasmic incompatibility factors and cytoplasmic hitchhiking across host populations

**DOI:** 10.1186/s12864-021-07906-6

**Published:** 2021-08-13

**Authors:** Jennifer L. Morrow, Markus Riegler

**Affiliations:** Hawkesbury Institute for the Environment, Western Sydney University, Locked Bag 1797, Penrith, NSW 2751 Australia

**Keywords:** Reproductive parasite, Haplotypes, Invasion dynamics, *cifA*, *cifB*, *Rhagoletis cerasi*, *Ceratitis capitata*

## Abstract

**Background:**

The endosymbiont *Wolbachia* can manipulate arthropod reproduction and invade host populations by inducing cytoplasmic incompatibility (CI). Some host species are coinfected with multiple *Wolbachia* strains which may have sequentially invaded host populations by expressing different types of modular CI factor (*cif*) genes. The tephritid fruit fly *Rhagoletis cerasi* is a model for CI and *Wolbachia* population dynamics. It is associated with at least four *Wolbachia* strains in various combinations, with demonstrated (*w*Cer2, *w*Cer4), predicted (*w*Cer1) or unknown (*w*Cer5) CI phenotypes.

**Results:**

We sequenced and assembled the draft genomes of the *Wolbachia* strains *w*Cer1, *w*Cer4 and *w*Cer5, and compared these with the previously sequenced genome of *w*Cer2 which currently invades *R. cerasi* populations. We found complete *cif* gene pairs in all strains: four pairs in *w*Cer2 (three Type I; one Type V), two pairs in *w*Cer1 (both Type I) and *w*Cer4 (one Type I; one Type V), and one pair in *w*Cer5 (Type IV). *Wolbachia* genome variant analyses across geographically and genetically distant host populations revealed the largest diversity of single nucleotide polymorphisms (SNPs) in *w*Cer5, followed by *w*Cer1 and then *w*Cer2, indicative of their different lengths of host associations. Furthermore, mitogenome analyses of the *Wolbachia* genome-sequenced individuals in combination with SNP data from six European countries revealed polymorphic mitogenome sites that displayed reduced diversity in individuals infected with *w*Cer2 compared to those without.

**Conclusions:**

Coinfections with *Wolbachia* are common in arthropods and affect options for *Wolbachia*-based management strategies of pest and vector species already infected by *Wolbachia*. Our analyses of *Wolbachia* genomes of a host naturally coinfected by several strains unravelled signatures of the evolutionary dynamics in both *Wolbachia* and host mitochondrial genomes as a consequence of repeated invasions. Invasion of already infected populations by new *Wolbachia* strains requires new sets of functionally different *cif* genes and thereby may select for a cumulative modularity of *cif* gene diversity in invading strains. Furthermore, we demonstrated at the mitogenomic scale that repeated CI-driven *Wolbachia* invasions of hosts result in reduced mitochondrial diversity and hitchhiking effects. Already resident *Wolbachia* strains may experience similar cytoplasmic hitchhiking effects caused by the invading *Wolbachia* strain.

**Supplementary Information:**

The online version contains supplementary material available at 10.1186/s12864-021-07906-6.

## Background

Maternally inherited endosymbiotic *Wolbachia* bacteria (Alphaproteobacteria) of arthropods can affect host reproduction and fitness, including host immunity, in a multitude of ways [1, 2], and consequently, influence the diversity of mitochondria [3] and possibly other cytoplasmic and non-cytoplasmic factors [4]. One commonly reported reproductive manipulation by *Wolbachia* is cytoplasmic incompatibility (CI). In its simplest form, CI involves a modification to the sperm of a *Wolbachia*-infected male which is not rescued after fertilisation of an egg from an uninfected female, resulting in embryonic mortality [5]. In contrast, an infected female transmits *Wolbachia* to her eggs thereby restoring successful embryonic development. Other reproductive manipulations by *Wolbachia* are male killing (MK), thelytokous parthenogenesis and feminisation [1]. While reproductive manipulations such as CI and MK bestow a reproductive advantage on *Wolbachia*-infected females in populations of mixed infection status, other *Wolbachia* effects can also contribute to host fitness. These can be beneficial such as nutrient provisioning [6, 7], increased fecundity [8] and pathogen protection [9, 10]; or costly such as reduced fecundity [11] and shortened life span [12].

A large number of host species are associated with more than one CI-inducing *Wolbachia* strain [13–15], indicating that some have experienced either consecutive or simultaneous invasions by multiple *Wolbachia* strains. These can occur as coinfections in individuals or as different infection types within and between populations and may result in complex interactions of CI and host fitness effects. For example, two or more CI-inducing *Wolbachia* strains co-infecting individuals of a species can produce patterns of unidirectional CI when one of these CI-inducing strains is missing in females, whereas males without this strain are still compatible with either type of infected females [16]. The rarer observed form is bidirectional CI which occurs when two or more CI-inducing *Wolbachia* strains do not co-occur in the same individuals, and this can result in bidirectional reproductive barriers between differently infected populations contributing to reproductive isolation and speciation processes [17–19].

The genetic basis of CI has recently been uncovered with the finding that *Wolbachia* induces and rescues CI through the dual expression of the cytoplasmic incompatibility factor genes *cifA* and *cifB* located in *Wolbachia* prophage regions [20, 21]. A proposed two-by-one model predicts that both *cifA* and *cifB* induce CI, whereas *cifA* only is required for rescue [22]. Sequence similarity analyses have revealed a large diversity of *cif* genes in *Wolbachia* genomes with zero to four complete pairs of *cif* genes common in strains [23–25]; but up to seven *cif* complexes have been found in some strains which also include unpaired, partial or non-functional *cif* genes [26]. The diversity of *cifA* and *cifB* gene products are classified as Type I to Type V based on amino acid similarity in functional domains [20, 23, 27]. The CI phenotype has been demonstrated by transgenic expression for Type I, Type II and Type IV *cif* genes [20, 21, 28], while *w*No, with a single Type III *cif* gene pair, and *w*Stri, with multiple Type V *cif* gene pairs, both also induce CI [27, 29–31]. Closely related *cif* genes within a type tend to be compatible; this applies particularly to *cifA* genes, whereas more variability in *cifB* genes correlates with phenotypic variation [28, 32]. However, divergence across the different types results in incompatibility between *cif* genes of Type I (e.g. in *w*Mel and *w*Pip), Type II (e.g. in *w*Ri) and Type IV (e.g. in *w*Pip) [20, 21, 28]. Therefore, the diversity and modularity of *cif* genes found in and across *Wolbachia* genomes may explain the complexity of CI interactions seen between *Wolbachia* strains, including the expression of bidirectional CI between strains with different types and/or numbers of *cif* genes even if these strains have similar multi locus sequence typing (MLST) profiles [33].

With their seminal paper on the incompatible populations of the European cherry fruit fly, *Rhagoletis cerasi* (Tephritidae), Boller and Bush [34] unknowingly established a key study system for *Wolbachia* population dynamics and CI. Their findings fit the model of unidirectional CI expressed between southern and northern populations of this species [35], which was hypothesised to be induced by intracellular *Rickettsia*-like microorganisms identified by electron microscopy [36]. Later, two *Wolbachia* strains, *w*Cer1 and *w*Cer2 were discovered that existed as either single *w*Cer1 infections in all individuals of all populations, or coinfections with *w*Cer2 in almost all individuals of southern populations, with individuals of transitional populations between the two population blocks displaying either of the two infection types [13, 37]. The strains’ geographic distribution correlated with the patterns of the previously reported unidirectional CI thereby indicating that *w*Cer2 induces CI between these populations which *w*Cer1 did not rescue [13]. The interactions of *R. cerasi* with *Wolbachia* were further complicated by the discovery of three other strains, usually found at lower titres than *w*Cer1 and *w*Cer2 [14]. Of the five strains, *w*Cer1, *w*Cer2, *w*Cer4 (all supergroup A strains) and *w*Cer5 (a supergroup B strain) were characterised by MLST [38], however, the existence of the strain *w*Cer3 was unclear because it was only ever detected as a *wsp* gene sequence by molecular cloning of *wsp* PCR amplicons, and consisted of a sequence which was a recombinant between *wsp* of *w*Cer2 and *w*Cer5 [14]. Across the host range *w*Cer3 was rare, whereas the prevalences of *w*Cer4 (60–78%) and *w*Cer5 (3–100%) were moderate and without any clear patterns [14] when contrasted with the distribution of *w*Cer1 and *w*Cer2 [13, 37]. Therefore, co-infections of *R. cerasi* individuals can include all possible combinations of *w*Cer1 with one, two or three of the strains *w*Cer2, *w*Cer4 and *w*Cer5 [14, 38].

While there is strong indirect and correlative evidence for the CI phenotype of *w*Cer2 in *R. cerasi* because of the distribution of *Wolbachia* strains and CI patterns [13, 14], the direct testing of CI phenotypes of the *Wolbachia* strains in this host species by crossing experiments between individuals of defined infection status is difficult due to its strict univoltine life cycle with an obligate pupal diapause [39] and complex laboratory rearing protocols [40]. However, the capacity of *w*Cer2 and *w*Cer4 to induce and rescue CI was demonstrated in a series of experiments involving transfer into novel host species by microinjections: for *w*Cer2 this resulted in expression of moderate CI in *Drosophila simulans* [41], and complete CI in the Mediterranean fruit fly *Ceratitis capitata* [42] and the olive fly *Bactrocera oleae* [43]; for *w*Cer4 it resulted in the expression of complete CI in *C. capitata* [42]. Moreover, whole genome sequencing of *w*Cer2 from infected *R. cerasi*, *D. simulans* and *C. capitata* revealed that the *w*Cer2 genome contains three pairs of Type I *cif* genes and one pair of Type V *cif* genes [24, 44].

Furthermore, there is a tight linkage of *w*Cer2 with a particular mitochondrial haplotype of *R. cerasi*, denoted haplotype 2 (HT2) which differs by a single nucleotide polymorphism (SNP; a synonymous third codon transition) in the mitochondrial *cytochrome oxidase subunit I* (COI) gene from HT1 found in individuals lacking *w*Cer2 [45]. This suggests mitochondrial hitchhiking of HT2 with a recent and still ongoing CI-driven invasion of host populations by *w*Cer2 [13, 37, 46, 47], and this was expected as a consequence of an ongoing *Wolbachia* invasion [3]. Besides this pattern of mitochondrial hitchhiking, it also appears that overall *R. cerasi* has very low mitochondrial DNA diversity (i.e. just two COI haplotypes) which may be indicative of several consecutive selective sweeps of mitochondrial genomes which has resulted in the elimination of mitogenome diversity in this species because of repeated invasions by *Wolbachia*. Specifically, *w*Cer1 which is fixed across *R. cerasi* populations is tightly linked with HT1, and may have invaded this host by CI, prior to the host’s invasion by *w*Cer2 [45]. A non-exclusive alternative reason for its high prevalence could be that *w*Cer1 provides a fitness benefit to the host, but this could still cause a selective sweep of an associated haplotype [3]. Furthermore, while *w*Cer4 causes CI in the novel host *C. capitata* [42], the CI potential and invasion history of *w*Cer4 and *w*Cer5 in their native host *R. cerasi* remain unknown. These strains may also have invaded the host by CI, prior to the invasions by *w*Cer2 and *w*Cer1. Alternatively, they may have other mechanisms by which they have invaded and are maintained in host populations, and this could include MK [48, 49]. A MK candidate gene has recently been identified within the *Wolbachia* prophage WOMelB region of *D. melanogaster* in the vicinity of *cifA* and *cifB* and named *WO-mediated killing* (*wmk*). It has six additional orthologues in the *w*Mel genome, but *wmk* is almost identical to a single homologue in *w*Rec, the MK *Wolbachia* strain of *Drosophila recens*. *Wmk* can cause MK when highly expressed in transgenic *D. melanogaster*, while *wmk* and its orthologues in *w*Mel do not have this effect [50].

Here we sequenced and analysed the genomes of *w*Cer1, *w*Cer4 and *w*Cer5, and compared these with the previously sequenced *w*Cer2 genome [24] with a particular focus on their *cif* and *wmk* gene repertoires. We expected to find full sets of diverse *cif* genes: for *w*Cer4 because of its CI expression in the novel host *C. capitata*, and for *w*Cer1 because of its very high prevalence and mitochondrial diversity patterns in the native host *R. cerasi*. Furthermore, we expected different (and potentially fewer) *cif* gene pairs and types in *w*Cer1, *w*Cer4 and *w*Cer5 than found in *w*Cer2 which has more recently infected this host species. This is because for any CI drive to occur, newly arriving *Wolbachia* strains would require *cif* genes that are novel to a host species already infected by other resident *Wolbachia* strains. We did not have prior expectations with regard to the presence of *wmk* genes because MK in *R. cerasi* has not been reported. Furthermore, the expression of MK also strongly depends on the host genotype [51, 52].

Moreover, we explored the *R. cerasi* mitogenomes of the individuals from which we obtained the *Wolbachia* genomes and used these mitogenomic data to guide the extraction and analysis of additional mitochondrial and *Wolbachia* SNP data, *Wolbachia* infection status and geographic information from a published double digest restriction-site associated DNA sequencing (ddRADseq) dataset of 192 *R. cerasi* individuals from six European countries [53]. We expected that greater mitochondrial haplotype variation is found in HT1 individuals lacking *w*Cer2 because these would not have experienced the selective sweep of HT2 caused by the *w*Cer2 invasion. Similarly, we expected to find greater SNP variation within the genomes of *Wolbachia* strains (i.e. *Wolbachia* strain variants) that have a longer association with *R. cerasi* because their genomes would have had more time to acquire new mutations since host invasion. They could also have experienced cytoplasmic hitchhiking effects similar to the ones experienced by mitochondrial genomes due to the *w*Cer2 invasion. Finally, we combined these three approaches of data analyses, (i) *cif* gene diversity and module number, (ii) mitogenome variant analyses and (iii) *Wolbachia* strain variant analyses, to infer the historical order of *Wolbachia* strain invasions in *R. cerasi*. We anticipated finding confirmation that *w*Cer2 is the most recent invader in this host species, following the prior invasions by *w*Cer1 and the other strains.

## Results

### Gene content of the three *Wolbachia* genomes *w*Cer1, *w*Cer4 and *w*Cer5

**Fig. 1:**
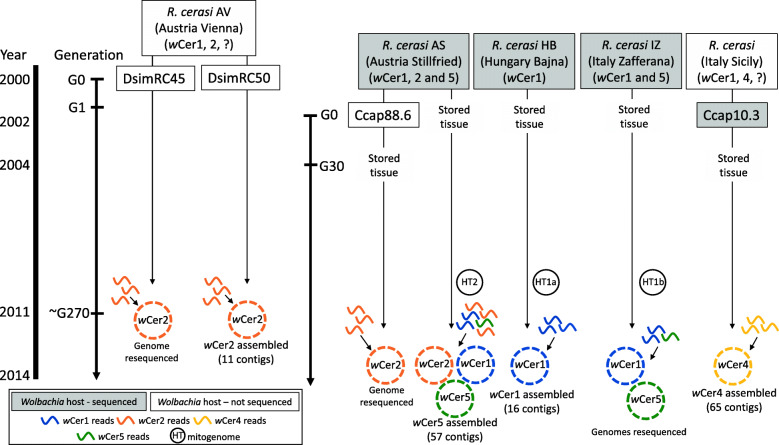
Schematic overview of the *Wolbachia* genomes (dashed circles) and mitochondrial genomes (closed circles) sequenced from four fruit fly individuals in this study (grey boxes). Genome sequences of *w*Cer2 have previously been obtained by Morrow et al. [24] from four host individuals (DsimRC45, DsimRC50, Ccap88.6, RcerAS). The timeline demonstrates when microinjected lines were established and at which generation post-injection individuals were sampled for genome sequencing

Genome amplification libraries of three *R. cerasi* field-collected individuals, one each from three geographically distant and genetically diverged populations, Austria (RcerAS), Hungary (RcerHB) and Italy (RcerIZ), and one individual of the microinjected *C. capitata* laboratory population (Ccap10.3) were sequenced to acquire the genomes of four *Wolbachia* strains and the *R. cerasi* hosts’ mitochondria (Fig. 1). Reads from each library were initially mapped to the MLST markers of each of the strains *w*Cer1, *w*Cer2, *w*Cer4 and *w*Cer5 to confirm the infection status of each individual used for library preparation (Table 1). RcerHB harboured *w*Cer1 only, and Ccap10.3 harboured *w*Cer4 only; RcerIZ was coinfected with *w*Cer1 and *w*Cer5, and RcerAS was coinfected with *w*Cer1, *w*Cer2 and *w*Cer5. None of the libraries contained the recombinant *wsp* gene of *w*Cer3.

**Table 1:** The mapping coverage of mitogenomes and genomes of *Wolbachia* strains obtained from three *Rhagoletis cerasi* individuals and one *Ceratitis capitata* individual. Genomes of *w*Cer1, *w*Cer2 and *w*Cer4 were assembled from libraries of individuals which only had one strain. Then these reference genomes were used to competitively map reads of RcerAS to *w*Cer1, *w*Cer2 and *w*Cer5, and reads of RcerIZ to *w*Cer1 and *w*Cer5; all other libraries (and sampled libraries) were mapped to a single *Wolbachia* strain. Subsampling of total reads was selectively applied to normalise the number of mapped reads for comparison between libraries. Mapping parameters were 97% similarity over 97% length, keeping only properly paired reads

Host Species	***Rhagoletis cerasi***			***C. capitata***
**Population**	RcerHB; Bajna, Hungary	RcerIZ; Zafferana, Italy	RcerAS; Stillfried, Austria	Ccap10.3 (WolMed S10.3)
**Tissue source (DNA extraction)**	single pupa (whole individual)	single larva (whole individual)	single pupa (whole individual)	single adult (abdomen)
***Wolbachia*strain detected by MLST markers**	*w*Cer1	*w*Cer1; *w*Cer5	*w*Cer1; *w*Cer2; *w*Cer5	*w*Cer4
**No. of paired reads after QC**	125,415,852	147,147,772	131,385,710	130,835,872
**No. paired reads mapped to mtDNA (full library)**	19,390,560	1,053,116	39,939,364	31,839,758
**Percentage mapped to mtDNA**	15.46%	0.72%	30.40%	24.34%
**No. paired reads mapped to mtDNA (no. subsampled)**	81,548 (530,000)	85,244 (12million)	81,608 (270,000)	na
**Percentage mapped to mtDNA**	15.46%	0.71%%	30.23%%	na
**No. paired reads mapped to*w*Cer1 (full library)**	3,586,116	258,194	1,411,170	na
**Percentage mapped to*w*Cer1**	2.86%	0.18%	1.08%	na
**No. paired reads mapped to*w*Cer1 (no. subsampled)**	286,212 (10million)	na	278,582 (26million)	na
**Percentage mapped to*w*Cer1**	2.86%	na	1.07%	na
**No. paired reads mapped to*w*Cer2 (full library)**	na	na	2,561,484	na
**Percentage mapped to*w*Cer2**	na	na	1.95%	na
**No. paired reads mapped to*w*Cer2 (no. subsampled)**	na	na	1,697,166 (87million)	na
**Percentage mapped to*w*Cer2**	na	na	1.95%	na
**No. paired reads mapped to*w*Cer4 (full library)**	na	na	na	1,701,414
**Percentage mapped to*w*Cer4**	na	na	na	1.30%
**No. paired reads mapped to*w*Cer5 (full library)**	na	51,402	191,892	na
**Percentage mapped to*w*Cer5**	na	0.03%	0.15%	na

The three new *Wolbachia* draft genomes presented here were not closed but deemed to be near complete by BUSCO analysis (Table 2). The BUSCO score for the *w*Cer1 genome (16 contigs) was 82.8%, the *w*Cer4 genome (65 contigs) was 83.3%, and the *w*Cer5 genome (57 contigs) was 81%, and all three were comparable to complete *Wolbachia* genomes that also had BUSCO scores between 81.4 and 83.7%.

**Table 2:** Genome characteristics, number of scaffolds and BUSCO scores (genome completeness) for *w*Cer1, *w*Cer4, *w*Cer5 (in bold) and the genomes of 16 reference strains (ordered by supergroups A and B, and then alphabetically)

Strain	Host	Supergroup	Accession No.	Genome size (bp)	Number of scaffolds	GC%	Predicted CDSs	tRNAs	rRNAs	BUSCO score
*w*Au	*Drosophila simulans*	A	LK055284	1,268,461	1	35.2	1276	34	1 of each	185 (83.7%)
*w*CauA	*Carposina sasakii*	A	NZ_CP041215	1,449,344	1	35.0	1442	34	1 of each	184 (83.3%)
***w*Cer1**	***Rhagoletis cerasi*(RcerHB)**	**A**	**JADCNC01000000**	**1,255,676**	**16**	**35.2**	**1196**	**34**	**1 of each**	**183 (82.8%)**
*w*Cer2	*Drosophila simulans* (DsimRC50)	A	SOZK01000000	1,325,568	11	35.2	1259	34	1 of each	184 (83.3%)
***w*Cer4**	***C. capitata*(Ccap10.3)**	**A**	**JADCND01000000**	**1,239,646**	**65**	**35.1**	**1214**	**34**	**1 of each**	**184 (83.3%)**
*w*Ha	*Drosophila simulans*	A	NC_021089	1,295,804	1	35.1	1235	34	1 of each	183 (82.8%)
*w*Irr	*Haematobia irritans irritans*	A	NZ_CP037426	1,352,354	1	35.3	1439	34	1 of each	184 (83.3%)
*w*Meg	*Chrysomya megacephala*	A	NZ_CP021120	1,376,868	1	34.0	1298	34	1 of each	182 (82.4%)
*w*Mel	*Drosophila melanogaster*	A	NC_002978	1267,782	1	35.2	1271	34	1 of each	184 (83.3%)
*w*Rec	*Drosophila recens*	A	NZ_JQAM01000000	1,126,656	43	35.1	1111	34	1 of each	181 (81.9%)
*w*Ri	*Drosophila simulans*	A	NC_012416	1,445,873	1	35.2	1396	34	1 of each	183 (82.8%)
*w*Suz	*Drosophila suzukii*	A	NZ_CAOU02000000	1,415,350	110	35.7	1321	34	1 of each	184 (83.3%)
*w*VitA	*Nasonia vitripennis*	A	NZ_MUJM01000000	1,211,929	142	35.1	1097	34	1 of each	185 (83.7%)
*w*AlbB	*A. albopictus*	B	NZ_CP031221	1,484,007	1	34.4	1418	34	1 of each	180 (81.4%)
***w*Cer5**	***Rhagoletis cerasi*(RcerAS)**	**B**	**JADCNE01000000**	**1,180,723**	**57**	**33.9**	**1091**	**34**	**1 of each**	**179 (81.0%)**
*w*Di	*Diaphorina citri*	B	CP051264	1,538,623	1	33.9	1418	34	1 of each	184 (83.3%)
*w*No	*Drosophila simulans*	B	NC_021084	1,301,823	1	34.0	1220	34	1 of each	184 (83.3%)
*w*Pip	*Culex quinquefasciatus*	B	NC_010981	1,482,455	1	34.2	1410	34	1 of each	181 (81.9%)
*w*Stri	*Laodelphax striatellus*	B	NZ_MUIX01000000	1,786,382	2	33.8	1747	34	1 of each	183 (82.9%)

**Fig. 2:**
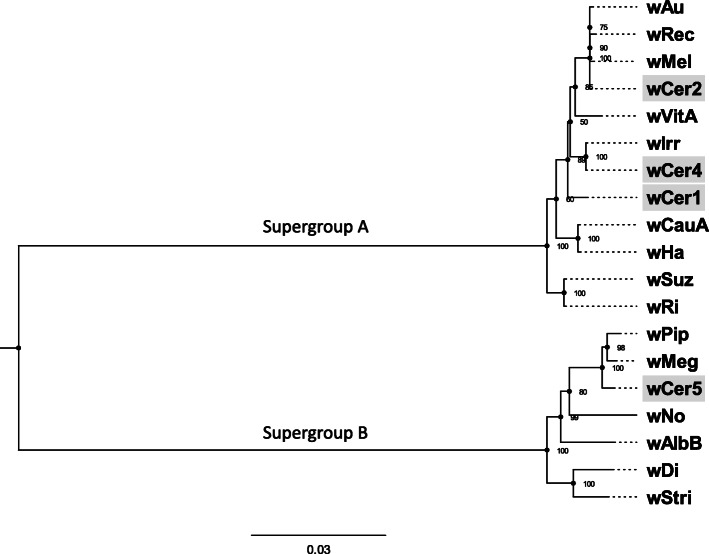
Maximum likelihood tree of *Wolbachia* genomes based on 256 orthologous genes. These genes are single-copy coding genes present in each of the 19 genomes and include 183,819 nucleotide positions of which 25,996 are parsimoniously informative. The general time reversible base substitution model (GTR + F + R2) was used to produce the tree, supported by 1000 bootstrap repetitions. *w*Cer genomes are shaded grey

OrthoFinder assigned 24,268 coding genes (97.6% of total 24,859 coding genes) of 19 *Wolbachia* genomes to 1373 orthogroups. Of these, 738 orthogroups were present in all genomes, and 664 consisted entirely of single-copy genes (Additional File 1). Testing for recombination using PhiPack identified 408 orthogroups that were excluded, with 256 orthogroups remaining. Testing of monophyly of the remaining genes for supergroup A and B strains (Table 2) found no additional genes that should be excluded due to polyphyly. A maximum likelihood tree was built on this set of 256 orthologous genes of 19 *Wolbachia* genomes and included 183,819 nucleotide sites of which 25,996 were parsimony-informative sites (Fig. 2). This analysis confirmed the assignment of *w*Cer1 and *w*Cer4 (along with *w*Cer2) into supergroup A and *w*Cer5 into supergroup B.

Visualisation of the intersection of orthologous genes using the UpSet graph also supported the strong differentiation of supergroup A and B genomes in terms of gene content (Additional File 1). The largest grouping included all the 19 genomes (738 orthogroups) but the next most abundant groups were exclusively the supergroup B strains (49 orthogroups) and supergroup A strains (28 orthogroups).

According to the maximum likelihood phylogenetic tree, *w*Cer1 was basal to the clade containing *w*Cer2 and *w*Cer4. *w*Cer4 was most closely related to *w*Irr (Fig. 2), sharing 6 unique orthogroups. Prophage regions were identified using PHASTER, with four regions in *w*Irr which cumulatively equalled 73.3 kb, while *w*Cer4 had two regions equalling 54.3 kb (Additional File 2). Similarly, *w*Cer1 had two prophage regions equalling 45.1 kb. It is possible that the fragmentation of the genome assemblies means that prophage regions that are split across contigs do not meet the threshold for identification. However, this was not supported by mapping of the reads from Ccap10.3 (*w*Cer4) and RcerHB (*w*Cer1) onto *w*Irr at 90% similarity and 60% length, which showed that there were many genes in the prophage regions of *w*Irr that were absent from *w*Cer4 and *w*Cer1. In comparison, *w*Cer2 had three annotated prophage regions, cumulatively equalling 170 kb [24], and, therefore, the largest prophage number and sequence length when compared to the other strains infecting this host species.

The *w*Cer5 genome was most closely related to *w*Pip and *w*Meg, confirming its placement in supergroup B (Fig. 2), with seven orthologous groups unique to these three, six unique to *w*Cer5 and *w*Meg, and three unique to *w*Cer5 and *w*Pip (Additional File 1). In comparison to the other strains *w*Cer5 had the smallest representation of prophage genes, with the presence of one incomplete region of 8.4 kb (Additional File 2).

### *Cif* and *wmk* genes in *w*Cer genomes

Orthology to verified *cifA* and *cifB* genes identified two pairs of Type I *cif* genes in *w*Cer1; one pair of Type I plus one pair of Type V *cif* genes in *w*Cer4; and one pair of Type IV *cif* genes in *w*Cer5 (Table 3). Original annotation of *w*Cer2 identified three pairs of Type I *cif* genes and a single Type V *cifB* gene [24], but reanalysis with OrthoFinder using six additional *Wolbachia* reference strains (particularly *w*StriCN and *w*Irr) improved the identification of Type V *cif* genes, and the hypothetical gene E3V96_3725 contiguous with the previously identified Type V *cifB* was annotated as *cifA*_*wCer2[T5]*._ Therefore, *w*Cer2 had four complete sets of *cif* genes and the largest number of *cif* modules in this host species.

**Table 3:** *cif* genes in *Wolbachia* strains of *Rhagoletis cerasi*

	Gene	Size (aa)	Locus	Orthologue, size (aa), % similarity	Type	Evidence for CI
***w*Cer1**	INQ25_05555	491	*cifA*	*w*Ha_RS01435, 492, 91%	I	UNCERTAIN: No crossing experiment data exists; but strong linkage with mitochondrial haplotype (HT1) in *Rhagoletis cerasi* field populations
	INQ25_05550	1143	*cifB*	*w*Ha_RS01430, 1148, 91%	I	
	INQ25_01115	491	*cifA*	*w*Ha_RS01435, 492,88%	I	
	INQ25_01120	1150	*cifB*	*w*Ha_RS01430, 1148, 92%	I	
***w*Cer2**	E3V96_03425	475	*cifA*	*w*Mel_RS02835, 475, 99%	I	YES: Experimental evidence for CI in multiple novel hosts - *Drosophila simulans*, *Ceratitis capitata*, *Bactrocera oleae*; strong linkage of *w*Cer2 with mitochondrial haplotype (HT2) in *R. cerasi* field populations
	E3V96_03430	1174	*cifB*	*w*Mel_RS06940, 1174, 99.7%	I	
	E3V96_02935	481	*cifA*	*w*VitA_RS00555, 499, 75%	I	
	E3V96_02940	1531	*cifB*	*w*VitA_RS00550, 1523, 85%	I	
	E3V96_06520	492	*cifA*	*w*Pip_RS01410, 504, 90%	I	
	E3V96_06515	921	*cifB*	*w*Pip_RS01415, 1175, 83%	I	
	E3V96_03725	438	*cifA*	*w*Irr_E0495_RS03300, 429, 66%	V	
	E3V96_03720	3405	*cifB*	*w*StriCN_BVG17_RS00730, 3083, 72%	V	
***w*Cer4**	INQ27_01280	494	*cifA*	*w*Ha_RS01435, 492, 79%	I	YES: Experimental evidence for strong CI in novel host *C. capitata*
	INQ27_01275	1166	*cifB*	*w*Pip_RS01415, 1175, 85%	I	
	INQ27_01270	415	*cifA*	*w*StriCN_BVG17_RS06595, 415, 84%	V	
	INQ27_01265	3332	*cifB*	*w*StriCN_BVG17_RS06590, 4358, 72%	V	
***w*Cer5**	INQ21_01080	446	*cifA*	*w*Pip_RS01460, 446, 99.8%	IV	UNCERTAIN: No crossing experiment data exists; but very high similarity of *cif* gene sequences to the Type IV *cifA/B* genes of *w*Pip with proven CI induction
	INQ21_01085	733	*cifB*	*w*Pip_RS01465, 733, 99.6%	IV	

**Fig. 3:**
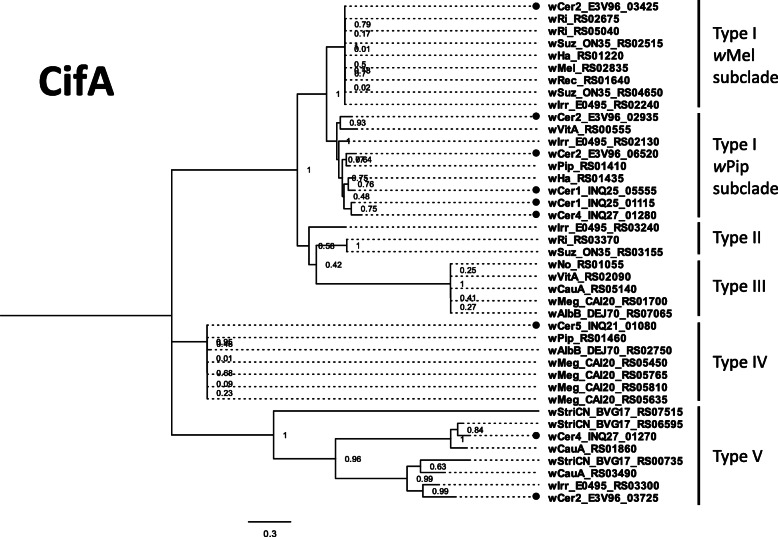
Maximum likelihood tree for *cifA* genes of 17 *Wolbachia* strains. The base substitution model TPM3 + F + G4 was used to produce the tree, supported by 1000 bootstrap repetitions. *cifA* genes from *w*Cer1, *w*Cer2, *w*Cer4 and *w*Cer5 are represented with black dots

**Fig. 4:**
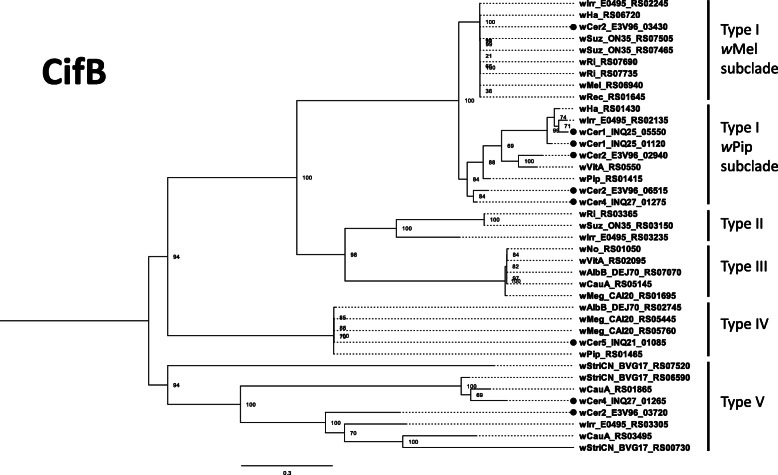
Maximum likelihood tree for *cifB* genes of 17 *Wolbachia* strains. The base substitution model TPM3 + F + I + G4 was used to produce the tree, supported by 1000 bootstrap repetitions. *cifB* genes from *w*Cer1, *w*Cer2, *w*Cer4 and *w*Cer5 are represented with black dots

The *cifA* maximum likelihood tree comprised 41 *cifA* orthologues, representative of all five types, and was built on an alignment of 1884 nucleotide sites, of which 1267 were parsimony-informative (Fig. 3; Additional File 3). The *cifB* gene alignment comprised 39 genes representative of all five types, with 5093 nucleotide sites of which 2651 were parsimony-informative (Fig. 4; Additional File 3).

**Fig. 5:**
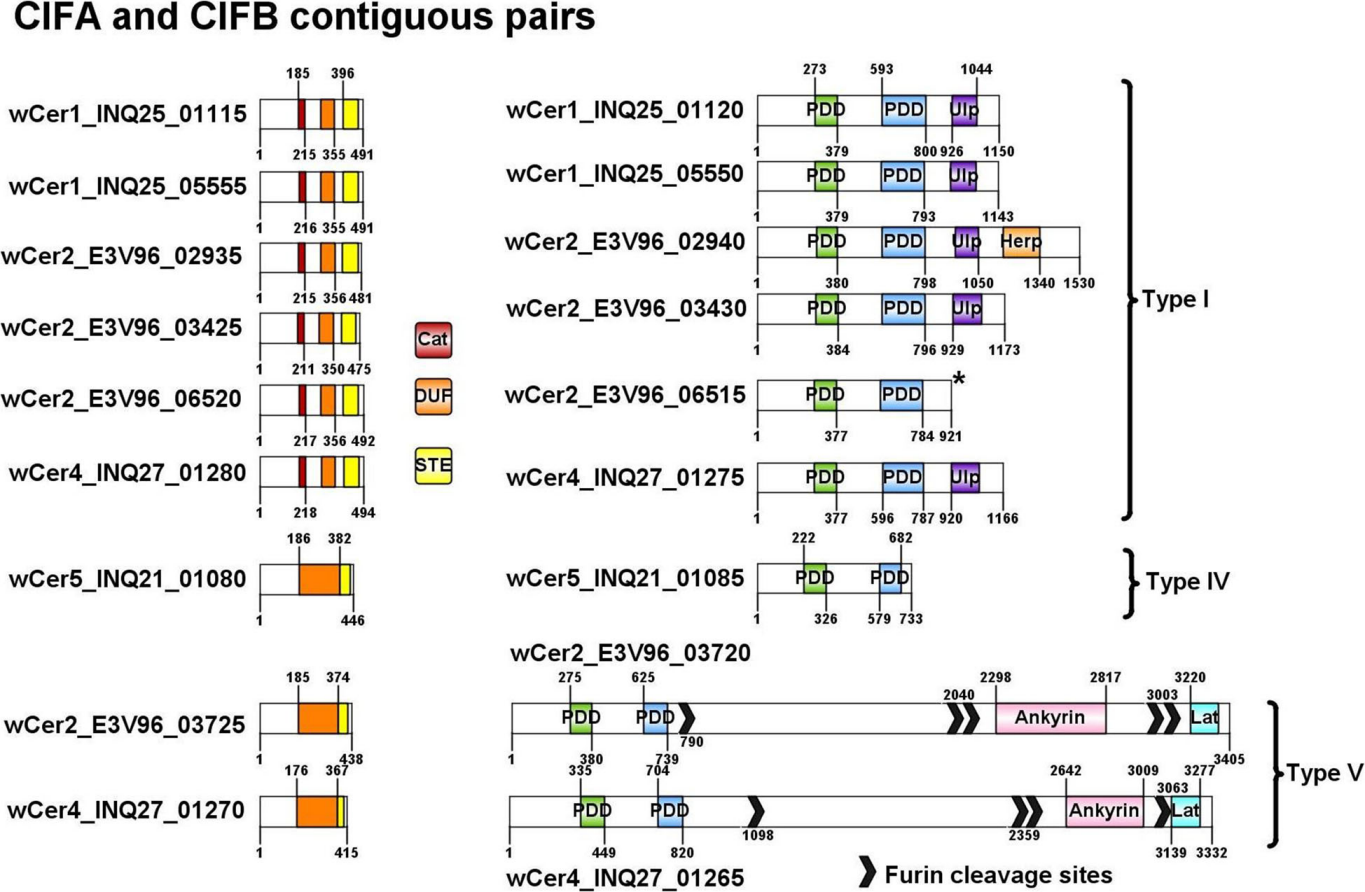
Protein domain structure of CifA and CifB of *w*Cer1, *w*Cer2, *w*Cer4 and *w*Cer5

Both contiguous *cif* gene pairs in *w*Cer1 were similar to the *cif*_*wPip[T1]*_ archetypes and contained complete functional domains and conserved amino acids of the Type I *cif* genes (Fig. 5). *w*Cer4 also contained complete and potentially functional *cif*_*wPip[T1]*_ -like genes, in addition to *cif*_*wCer4[T5]*_ genes where *cifB*_*wCer4[T5]*_ had a 2511 amino acid extension of ankyrin and latrotoxin domains and furin cleavage sites. This was similar to other Type V *cifB* genes including the *cifB*_*wCer2[T5]*_ gene E3V96_03720 [24], which also had an ankyrin and latrotoxin extension (Fig. 5). Both of these genes were similar in the PDDEXK nuclease domains to the Type IV and Type V *cifB* genes of *w*Pip and *w*StriCN respectively, notably to the conserved amino acids identified by Shropshire et al. [54]. *w*Cer5 is a supergroup B strain most closely related to *w*Pip and *w*Meg, containing a single contiguous pair of *cif*_*wCer5[T4]*_ genes that shared 99.8% (*cifA*) and 99.6% (*cifB*) amino acid similarity with CI inducing *cif*_*wPip[T4]*_.

Furthermore, we found a *w*Cer2 gene (E3V96_03405) with an identical amino acid sequence to *wmk* of *w*Mel (WD0626) which had previously been found to cause MK when highly expressed in transgenic *D. melanogaster*. No orthologues for this gene were found in *w*Cer4 and *w*Cer5. However, for a *wmk* homologue in *w*Mel, WD0508, for which transgenic expression did not alter sex ratios in *D. melanogaster*, orthologues were found in *w*Cer2 (similarity of 89%), *w*Cer4 (94%) and *w*Cer5 (93.6%). No orthologues of *wmk* or its homologues were found in *w*Cer1.

### Mitochondrial genome polymorphisms in different populations

**Fig. 6:**
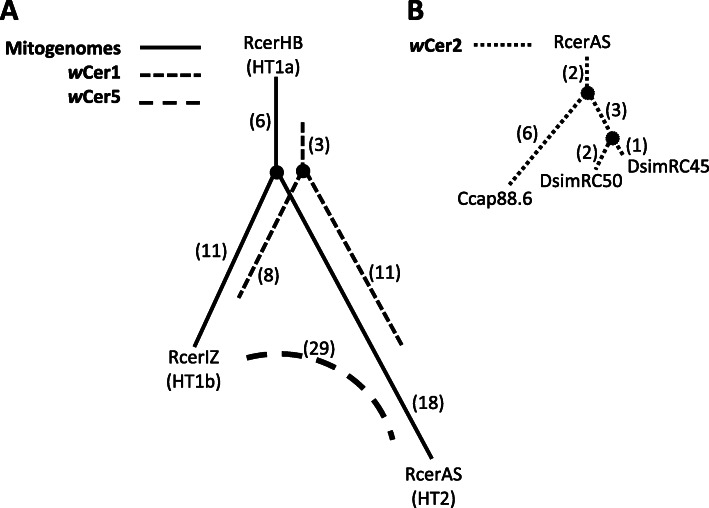
Haplotype networks for **(A)** mitogenome variants of RcerHB (HT1a), RcerIZ (HT1a) and RcerAS (HT2) and *Wolbachia* strains *w*Cer1, *w*Cer2 and *w*Cer5, and **(B)** *w*Cer2 variants between RcerAS and three novel host lineages (Ccap88.6, DsimRC45 and DsimRC50) infected by embryonic microinjection. Numbers (in parentheses) next to solid lines indicate SNPs between the mitogenome variants, and numbers (in parentheses) next to broken lines SNPs between the *Wolbachia* strain genome variants. Input alignments were based on library-specific consensus genomes (Additional Files 7, 8 and 9) determined by majority rule (nucleotide called when > 50% of mapped reads). *w*Cer4 was not included in this analysis because only one genome variant was sequenced

The mitochondrial genomes assembled from the three *R. cerasi* individuals were representatives of three (including geographically distant and genetically diverged) populations with different *Wolbachia* infection types (Additional File 4). Mitogenome comparisons revealed 17 SNPs and three indels in homopolymer regions between RcerHB and RcerIZ; 24 SNPs and five indels between RcerHB and RcerAS; and 29 SNPs and four indels between RcerIZ and RcerAS (Fig. 6). The two haplotypes HT1 and HT2 previously defined by one SNP difference (SNP position 2767 of the mitogenome) were found to be linked with *w*Cer1 and *w*Cer2, respectively. More specifically, the mitogenomes of the individuals RcerHB and RcerIZ were HT1 (denoted HT1a and HT1b, respectively) and these individuals did not have *w*Cer2, and the mitogenome of *w*Cer2-infected RcerAS was HT2 (Fig. 1).

A population genomic comparison of the level of mitogenome divergence within the two mitochondrial haplotypes was not possible due to the low sample replication of two HT1 mitogenomes and one HT2 mitogenome. However, mitochondrial haplotype differences were analysed and linked to *w*Cer2 presence or absence using a previously published ddRadseq dataset, which included 192 *R. cerasi* individuals from different locations with different *Wolbachia* strain combinations. Of the 41 differing sites (either SNPs or indels) between the three mitogenomes (Additional File 4), only 12 were represented by ddRadseq loci (Additional File 5). For HT1 individuals (without *w*Cer2), 8 of the 12 sites were polymorphic (32 individuals including 30 ddRadseq individuals plus RcerHB and RcerIZ); for HT2 individuals (with *w*Cer2) only 10 sites were represented by ddRADseq, and none was polymorphic (16 individuals including 15 ddRadseq individuals plus RcerAS). The variance in the Euclidean distance within the HT1 group of mitochondrial haplotypes (var = 0.07) was higher than group HT2 (var = 0.001). Furthermore, the PERMANOVA showed strong influence of the *w*Cer1/*w*Cer2 grouping on the distance measures between haplotypes (*p* = 0.001), but presence of *w*Cer4 (*p* = 0.736) or *w*Cer5 (*p* = 0.206) had no effect (Additional File 6).

### *Wolbachia* strain polymorphisms across populations

The variation across the *Wolbachia* strains independently isolated from geographically distant and genetically diverged host populations was investigated to infer the relative ages of the associations of *w*Cer1, *w*Cer2 and *w*Cer5 with *R. cerasi*. Consensus sequences (Fig. 6; Additional Files 7, 8 and 9) and variant information (Additional Files 10, 11 and 12) were extracted for each combination of strain and host population. Genome networks of complete consensus sequences for each strain, *w*Cer1 (RcerAS, RcerIZ, RcerHB), *w*Cer5 (RcerAS, RcerIZ) and *w*Cer2 (RcerAS, and three microinjected novel hosts *C. capitata* Ccap88.6, *D. simulans* DsimRC45, DsimRC50 [24]) showed that the numbers of SNPs between variants of strains across populations varied (Fig. 6), but no rearrangements or large gaps in sequence were noted. The number of SNPs in consensus sequences between the two *w*Cer5 variants (RcerAS, RcerIZ; 29 SNPs) was almost 60% higher than the number of SNPs between the two *w*Cer1 variants in the same two samples (RcerAS, RcerIZ; 19 SNPs). The consensus sequences for each strain for each individual were calculated by majority consensus with no lower coverage threshold, however the additional variant analysis also showed strain variation within host individuals. The *w*Cer1 of RcerHB showed at least two possible nucleotides (with minimum coverage of 35% of a given variant with minimum coverage threshold of 5 reads) at 16 sites; at 12 of these sites both nucleotides were present in the *w*Cer1 of RcerIZ, and at 10 sites both nucleotides were present in the *w*Cer1 of RcerAS. The overlap of variant sites in RcerIZ and RcerAS was at 11 sites (Additional File 10). The *w*Cer5 strain variation within each individual accounted for only four of the 29 SNPs between the *w*Cer5 variants of RcerAS and RcerIZ (Additional File 11). To ensure these results were not inflated by the collapse of non-identical multicopy genes into single genes in the draft genome, the sequence locations of the SNPs were determined (Additional Files 10 and 11). SNPs were found mostly in intergenic regions, single copy genes or multiple copy genes where *w*Cer1 or *w*Cer5 had similar orthologues to the reference genomes (based on *w*Cer1, *w*Au and *w*Mel genomes for *w*Cer1 SNPs; and *w*Cer5, *w*Meg and *w*Pip genomes for *w*Cer5 SNPs). However, our data indicated at most a single SNP in a transposase gene that was single copy in *w*Cer1 but multicopy in *w*Au and *w*Mel may be a false positive. For *w*Cer5, the single SNP in the phage gene patatin and nine of the 10 SNPs in the major tail sheath protein gene in RcerAS are true differences (> 58% frequency) from the reference sequence, while one heterozygous site may be a false positive. Notwithstanding these possible misassemblies, the number of changes still suggests *w*Cer5 has a greater number of SNPs than *w*Cer1 between RcerIZ and RcerAS.

For *w*Cer2, eight SNPs between the *Wolbachia* genome of Ccap88.6 and its donor RcerAS (Fig. 6) were detected; six or seven SNPs differentiated *w*Cer2 variants in two *D. simulans* hosts (DsimRC50 and DsimRC45) from their donor, but three SNPs were common to the two recipients, and an additional two were common to the Ccap88.6 and *D. simulans* lines (Fig. 6). All variants except one (for which 100% reads were different from the reference nucleotide) were found in the *w*Cer2 of RcerAS.

We also searched the ddRadseq reads mapped to the *Wolbachia* strains to find SNPs that would extend our dataset, and used the mapped reads to establish the infection status of each individual, but very few reads mapped to sites previously identified as polymorphic, and we were unable to extend analysis of *Wolbachia* strain genomic variation across more populations than those that were whole genome sequenced. However, the relative titre of the strains in each of the 46 ddRADseq samples which were informative was assessed using the mapping coverage of the ddRADseq reads on each *Wolbachia* strain at a minimum of five sites. Consistently *w*Cer2 had high coverage (>30x), *w*Cer1 had ~20x coverage, *w*Cer4 had ~2x coverage and *w*Cer5 had ~5x coverage, thereby confirming *w*Cer1 and *w*Cer2 as high titre infections, and *w*Cer4 and *w*Cer5 as low titre infections in this species.

The number of mutations between the *w*Cer1 variants and between the HT1 mitogenomes of RcerIZ and RcerHB (11 SNPs and 17 SNPs respectively), equated to 117x relatively more mutations in the mitogenome (*Wolbachia* genome is approximately 76x larger than the mitogenome). The *w*Cer1 genome comparison of RcerAS and RcerHB revealed 14 SNPs, the mitogenomes of those samples had 24 SNPs, which equated to 130x relatively more mutations in the mitogenome. The *w*Cer1 genome comparison of RcerAS and RcerIZ revealed 19 SNPs, the mitogenomes of those samples differed by 29 SNPs, i.e. 116x relatively more mutations in the mitogenome.

## Discussion

We sequenced and assembled three new *Wolbachia* strain genomes, *w*Cer1, *w*Cer4 and *w*Cer5, of the European cherry fruit fly, *R. cerasi*, and analysed these in conjunction with the previously sequenced genome of *w*Cer2 [24]. All genomes contained *cif* genes with functional domains which indicates CI is likely induced by all four strains. The larger number and diversity of prophage-associated *cif* gene modules, and lower *Wolbachia* strain and mitochondrial genome variant diversity associated with *w*Cer2 support the hypothesis that *w*Cer2 is the most recently acquired *Wolbachia* strain in this host species, while *w*Cer1 has been associated with *R. cerasi* for a longer period. The higher level of polymorphism between *w*Cer5 variants than between wCer1 variants in individuals of geographically distant populations suggests an even longer association of *w*Cer5 with *R. cerasi*. While the finding of *cif* genes with functional domains in *w*Cer1 together with its fixation in all *R. cerasi* populations are indicators of strong CI expression by this strain in this host species, this is less clear for *w*Cer4 and *w*Cer5 because of their patchier geographic distribution and more moderate prevalence in host populations when compared to *w*Cer1 and *w*Cer2 [14], without any clear linkage of *w*Cer4 and *w*Cer5 to mitochondrial haplotypes. However, *w*Cer4 causes strong CI in *C. capitata* [42], and *w*Cer5 is fixed in some populations of *R. cerasi* [14]. The loss of any linkage of *w*Cer4 and *w*Cer5 with a particular mitochondrial haplotype could indicate that these two strains colonised *R. cerasi* prior to the invasion by *w*Cer1 and *w*Cer2, and their lower prevalence and/or patchier distribution may indicate that their CI phenotype may be (i) weak (due to their lower titre), (ii) partially supressed by *R. cerasi* (as seen for *w*Mel in *Drosophila melanogaster* [55, 56]), or alternatively, (iii) *w*Cer1 and/or *w*Cer2 may be able to rescue some of the CI induced by *w*Cer4. The latter is unlikely for *w*Cer5, because its Type IV *cif* gene pair is unique in this host system. Furthermore, while the genome analyses revealed high similarities to the *wmk* genes of *w*Mel and *w*Rec in *w*Cer2, it is unlikely they cause MK in this host system, also because sex ratio biases have so far not been reported. Future research should investigate whether *w*Cer4 and *w*Cer5 are indeed maintained in populations by CI, and if so, how their variable prevalences found across populations affect the ongoing invasion of *R. cerasi* by *w*Cer2. Alternatively they are maintained because of beneficial host effects as seen for non-CI or weak CI-inducing strains in some host species [55, 57, 58]. An increased knowledge base surrounding the interactions of multiple CI-inducing *Wolbachia* strains in the same host species (including after artificial introduction by microinjection) is crucially important in *Wolbachia*-based management of pest and vector populations which are already infected by *Wolbachia* (e.g. [59, 60]).

### *Cif* and *wmk* genes in multiple co-occurring *Wolbachia* strains

When comparing the *cif* gene repertoires of the *Wolbachia* strains, *w*Cer1 has two intact pairs of *cif* genes, similar to the *cif*_*wPip[T1]*_ genes that recapitulate CI [21]; *w*Cer2 has two Type I *cif* gene pairs also in the *w*Pip Type I sub-clade with an additional Type I *cif* gene pair, almost identical to the archetypal *cif*_*wMel[T1]*_ gene pair [20, 24], and a *cif*_*wCer2[T5]*_ gene pair for which only *cifB* had previously been annotated [24]. All Cif proteins in *w*Cer1 (two pairs) and *w*Cer2 (four pairs) appear to have necessary functional domains, so, while there is no evidence from crossing experiments, *w*Cer2 may have the potential to rescue *w*Cer1-induced CI. This is most likely via the two closely related *cifA* from the *cifA*_*wPip[T1]*_ subclade, however the *w*Mel-like Type I *cifA* gene could also play a role. Such a prediction is supported by the demonstrated CifA rescue of CI induced by a similar but non-cognate CifA/B pair [28]. Conversely, the presence of multiple *cif* genes of the same type may cause additional CI and/or strengthen CI levels.

Furthermore, *cif*_*wCer2[T5]*_ may induce CI that is not rescued by Type I *cif* due to their dissimilarity. While there is no experimental evidence for CI induction by *cif*_*wCer2[T5]*_, Type V *cif* genes have characteristics of other *cif* types: (i) the *cifA and cifB* genes are adjacent, transcribed in the same direction and located in prophage regions; (ii) the domain structure is similar to *cif*_*wPip[T4]*_ (Fig. 5), which has been determined to recapitulate CI in a transgenic system [21]; and (iii) potential functionality of Type V *cif* genes is demonstrated in the CI-inducing strain *w*Stri which only contains Type V *cif* genes [26, 27]. However, to unequivocally discern the contribution to CI of individual *cif* pairs expressed in a *Wolbachia* strain containing multiple copies, transgenic expression of single *cif* genes or gene pairs is necessary.

Furthermore, *w*Cer4 induces CI and is bidirectionally incompatible with *w*Cer2 in the novel host *C. capitata* [42]. We found that *w*Cer4 had one pair of Type I *cif* genes encoding intact functional domains and were phylogenetically grouped in the *cifB*_*wPip[T1]*_ subclade. *w*Cer4 also had a Type V *cif* gene pair which was most similar to *cif*_*wStriCN[T5]*_ (84 and 72% amino acid similarity respectively) but only had 36 and 45% similarity with *cif*_*wCer2[T5]*._ Both *cif* gene pairs are potentially responsible for CI in *w*Cer4 and one or both are presumably incompatible with the *w*Cer2 *cif* gene pairs. The substantial divergence between the Type V *cif* genes in *w*Cer2 and *w*Cer4 suggests that they are likely incompatible. The repertoire of *cif* genes in *w*Cer4 also indicates this strain may be incompatible with *w*Cer1, due to the absence of Type V *cif* genes in *w*Cer1.

While it has not been demonstrated in crossing experiments that *w*Cer5 can induce CI, the genome sequencing of *w*Cer5 showed a high similarity of *cif*_*wCer5[T4]*_ to CI-inducing *cif*_*wPip[T4]*_ (over 99.6% amino acid identity), and is therefore likely to induce CI. Furthermore, *w*Cer5 is the only *w*Cer genome with Type IV *cif* genes in this host species, and unlike the other *w*Cer genomes has no Type I *cif* genes. Therefore, *w*Cer5 is likely bidirectionally incompatible with *w*Cer1, *w*Cer2 and *w*Cer4, and this could cause issues, e.g. slowing down of a *w*Cer2 invasion between populations that are polymorphic in infection status. One would expect that for invasion to be successful either *w*Cer2 and *w*Cer5 spread should be linked, or *w*Cer5 should already exist at a high enough prevalence in populations that are being invaded.

We have also investigated whether the *w*Cer genomes contain orthologues of the *wmk* gene sequence which can simulate a MK phenotype in transgenic *D. melanogaster* [50]. While *w*Cer2 has a gene with identical amino acid sequence, the other strains only have similarities to a *wmk* homologue which does not simulate MK. Furthermore, no sex ratio distortion has previously been observed in *R. cerasi* and novel hosts of *w*Cer2 and *w*Cer4. Therefore, it is unlikely MK is expressed in this host system.

### Incomplete self-rescue or fitness costs of *w*Cer2 and *w*Cer4 in novel hosts

An unresolved aspect to the phenotype of *w*Cer2 and *w*Cer4 in novel hosts *D. simulans* [41], *C. capitata* [42] and *B. oleae* [43] involves the reduced hatch rate seen in crosses involving parents that are infected with the same strain. This phenotype may be attributed to a fitness cost independent of CI, such as is associated with high *Wolbachia* titre [61], or incomplete self-rescue because uninfected control crosses and crosses between uninfected males and *w*Cer2 or *w*Cer4 infected females had significantly lower embryonic mortality.

We previously annotated a single unpaired Type V *cifB* gene in *w*Cer2, which we hypothesised either (i) caused lethality by toxic expression; (ii) induced weak CI when combined with a non-cognate *cifA* gene; or (iii) induced CI that was only partially rescued through activity of a non-cognate *cifA* gene [24]. However, reanalyses found that this *cifB* gene was not unpaired, and its relatively more diverged *cifA* partner has now been annotated due to the addition of more diverse *cif* gene types in the comparative genome analysis. Similarly, we also identified additional Type V *cifA* genes in *w*Irr [25] and *w*CauA which also exemplify the substantial diversity within the Type V clade.

While unpaired *cifB* genes are not involved in incomplete self-rescue, toxicity has been demonstrated in transgenic insects for *cifB*_*wRec[T1]*_ [28] and *cifB*_*wPip[T4]*_ [62] when the *cifB* transgene is expressed alone in males. Furthermore, *cifB*_*wRec[T1]*_ cannot be rescued by either cognate *cifA* or non-cognate *cif*_*wMel[T1]*_, so lethality is not strictly through CI [28], and this effect may only be indirectly related to CI competence. Incomplete self-rescue has not been reported for either *Wolbachia* strains *w*Rec or *w*Pip, so the previously mentioned results were obtained through sole transgenic expression of *cifB*. However, different expression levels in a natural situation may emulate this outcome. Therefore, expression studies that compare individual transcription levels of the *cif* gene repertoire of *w*Cer2 and *w*Cer4 may resolve this question. Similar research should also be done with *w*Tei, another strain that experiences incomplete self-rescue upon its transfer from its original host *Drosophila teissieri* to *D. simulans* [63].

### *w*Cer1 is fixed in *R. cerasi*, but *w*Cer5 has a longer host association

Based on consensus sequence comparisons, the divergence between *w*Cer5 variants is larger than the divergence between the respective *w*Cer1 variants (by 60%). This indicates that *w*Cer5 has been associated with *R. cerasi* for a relatively longer period than *w*Cer1. Furthermore, the variant analysis showed that strain variants comprised a polymorphic population. For *w*Cer5 there were few variable site overlaps, and therefore more accrued (or real) differences between the two sequenced individuals; in contrast, there were many variable site overlaps for *w*Cer1 variants of the same two individuals. The linkage of *w*Cer1 with HT1 is a clear indication of a more recent invasion of *w*Cer1 than *w*Cer5. No such link for *w*Cer5 with a mitochondrial haplotype was detected, and this could indicate that a previously existing linkage may have broken down as a consequence of the *w*Cer1 invasion in *R. cerasi*. *w*Cer5 maintains high prevalence in some *R. cerasi* populations where it routinely co-occurs with *w*Cer1, *w*Cer2 and *w*Cer4 (Additional File 5), but is low or absent in other populations [14]. It was consistently detected at low coverage in the WGS reads and ddRADseq suggesting low titre in this host. Its patchier distribution, however, suggests it did not invade all populations, or has been lost from some populations, which may be due to incomplete transmission or high fitness costs. Low titre infections may reduce transmission success [64], or cause weak or ineffective CI [65, 66]. Low titre of strains may be attributed to competition with other *w*Cer strains, diapause effects [16, 67], male age or male development time [55, 56], but evidence for this will be required from experiments that directly test transmission, CI and fitness costs of *w*Cer5.

### Effects of *w*Cer2 invasion on mitogenome and *Wolbachia* variant diversity

It has been demonstrated that *w*Cer2 is currently invading *R. cerasi* populations [13, 46], and this has caused a mitochondrial selective sweep [37, 47], previously only characterised by a single nucleotide difference in the COI gene [45]. We have revealed more mitogenomic variation between the genome sequenced individuals and extended the analysis using ddRADseq reads of 46 individuals from a large geographic area. We did not find any detectable mitogenomic variation in any of the 10 informative sites of individuals with *w*Cer2, whereas mitogenomic variation was detected in 8 of the 12 informative sites across individuals with HT1 haplotypes and without *w*Cer2. While mitochondrial network analysis could not be rooted with uninfected haplotypes because *w*Cer1 is fixed in this species, our data showed two-thirds of the variability between HT1 and HT2 haplotypes was found within the HT1 mitogenomes. This variability at multiple nucleotide sites within HT1 supports the previously detected mitochondrial hitchhiking due to the *w*Cer2 invasion [37], and suggests that HT1 variability has been acquired by *R. cerasi* since invasion by *w*Cer1.

Besides the mitochondrial sweep caused by a CI-driven invasion of a *Wolbachia* strain, it is also expected that any co-infecting strain already resident in host populations will also experience a selective sweep. For *R. cerasi*, such cytoplasmic hitchhiking is expected to be seen for *w*Cer1, *w*Cer4 and *w*Cer5 associated with the invading *w*Cer2 strain, and will result in loss of accumulated intrastrain diversity across populations, whereas *w*Cer1, *w*Cer4 and *w*Cer5 not associated with *w*Cer2 will maintain any original intrastrain diversity. While we found SNPs within *w*Cer strain genomes, due to insufficient coverage from the ddRadseq dataset, no additional information could be extracted to test for cytoplasmic hitchhiking of *w*Cer1, *w*Cer4 and *w*Cer5. However, the newly established genome data provide the basis for such investigations in the future.

Previously it was thought, based on identical MLST profiles of *w*Cer2 and *w*Cin2 of the North American eastern cherry fruit fly *Rhagoletis cingulata* which has more recently become invasive in Europe, that *w*Cer2 may have been acquired by *R. cerasi* from *R. cingulata*. However, this was disproven as, besides the MLST genes, the genomes of *w*Cer2 and *w*Cin2 are fairly distinct from each other [44]. However, while the origin of *w*Cer2 in *R. cerasi* is still unclear, our mitogenome diversity analyses still suggest a fairly recent introduction of *w*Cer2 to *R. cerasi*. In contrast, the high variation in mitochondrial haplotypes associated with *w*Cer1 suggests a longer association with *R. cerasi*. The evidence previously presented that *w*Cer1 is the source of the horizontal acquisition of *w*Cin1 in *R. cingulata* as part of its colonisation of Europe [68] will still need further validation by sequence analysis of the *w*Cin1 genome and comparison with the *w*Cer1 genome presented here.

## Conclusions

Our analyses of the four *Wolbachia* genomes *w*Cer1, *w*Cer2, *w*Cer4 and *w*Cer5 have provided insights into the diversity and modularity of *cif* gene interactions in the multiply infected host species *R. cerasi*. Next, more directed studies should be performed to investigate the capacity of each *cif* gene module in *w*Cer genomes to induce and rescue CI, understand the interaction of multiple *cif* gene modules when expressed in the same strain (such as for *w*Cer1, *w*Cer2 and *w*Cer4) or multiple strains in a single host, and resolve the fitness costs (toxicity or incomplete CI self-rescue) that have been demonstrated for single infections of *w*Cer2 and *w*Cer4 in novel hosts. Furthermore, mitogenomes and *Wolbachia* genomes from WGS projects can guide and increase resolution of SNP analyses from reduced representation genomic datasets, such as ddRadSeq. This enabled us to link *Wolbachia* strain infection with mitogenome haplotypes in individuals and clearly demonstrated haplotype variation associated with *Wolbachia* infections and the more recent acquisition of *w*Cer2.

## Methods

### Source of individuals for genome sequencing

One individual was sourced for genome sequencing from each of three populations of *R. cerasi*: RcerAS from Stillfried, Austria (approximately 40 km NE of Vienna), collected in 2001; RcerIZ from Zafferana in eastern Sicily, Italy, collected in 2001; and RcerHB from Bajna, Hungary (approximately 40 km NW of Budapest), collected in 2000 (Fig. 1). The geographic distances between sites ranged from 200 to 2000 km, with a high host genetic divergence of *R. cerasi* between Sicily and the two central European sites [53]. Based on *Wolbachia* strain-specific PCR, individuals from Stillfried carried both *w*Cer1 and *w*Cer2 while the individuals from Zafferana and Bajna carried *w*Cer1 without *w*Cer2 [13]. Furthermore, individuals from these populations may also carry *w*Cer3, *w*Cer4 and *w*Cer5 [14, 38].

In 2002, *w*Cer4 was successfully established in an isofemale line of *C. capitata* Benakeion (WolMed S10.3, hereafter called Ccap10.3) by microinjection, using a donor population of *R. cerasi* from Sicily [69]. One Ccap10.3 individual from generation 30 was selected for DNA extraction and whole genome sequencing.

Previously, the genome of *w*Cer2 [24] was assembled from sequencing reads derived from embryos of *D. simulans* isofemale line DsimRC50 carrying a single infection of *w*Cer2, that had been established following embryonic microinjection from *R. cerasi* individuals from Schoenbrunn, Vienna [41]. The *w*Cer2 genome was also obtained from another *D. simulans* isofemale line (DsimRC45), the RcerAS individual, and from an individual of another *C. capitata* isofemale line, Ccap88.6, which was established after microinjection of *w*Cer2 from RcerAS individuals into *C. capitata* Benakeion individuals [24].

### DNA extraction and high-throughput DNA sequencing

For *R. cerasi* and *C. capitata*, DNA was extracted from an individual larva (RcerIZ), pupa (RcerAS and RcerHB) or adult female abdomen (Ccap10.3), based on availability of source material. The samples were first surface sterilized by immersion in 5% sodium hypochlorite for 1 min, followed by rinsing in triton-x and multiple washes of water. The QiaAmp DNA kit was used to isolate genomic DNA from each sample, according to the manufacturer’s instructions, including RNase treatment, with the exception that the final elution was using 50 μL of nuclease-free water. Quality of genomic DNA was checked by gel electrophoresis. Whole genome amplification of 5 to 20 ng genomic DNA using the Qiagen Repli-G midi kit was performed to increase the quantity and proportion of bacterial DNA in the sample. The amplified DNA was cleaned again using the QiaAmp kit and eluted in 50 μL nuclease-free water.

The quality and yield of all DNA samples was ascertained by gel electrophoresis, Nanodrop spectroscopy and Qubit double-stranded DNA quantification system. Libraries for each sample were prepared with TruSeq PCR-free (350 bp insert) library kit (Illumina), using 1 μg of input DNA, and the paired-end (2 × 125 bp) libraries were sequenced on an Illumina HiSeq 2500 platform (NGS Facility, Western Sydney University).

### Bioinformatics

The bioinformatics pipeline was implemented as described in Morrow, et al. [24], with minor modifications. CLC Genomics Workbench ver.12 (Qiagen) was used for quality control, de novo assembly, mapping and variant calling. In order to choose the best sample library to use for the assembly of each of the *Wolbachia* genomes *w*Cer1, *w*Cer4 and *w*Cer5, the trimmed reads for each library were mapped at 100% similarity to the *wsp* gene and the five MLST sequences (*gatB*, *coxA*, *hcpA*, *fbpA* and *ftsZ*) that were previously obtained for these strains [14]. As the MLST profile for *w*Cer3 is unknown [38] we used the *w*Cer3 *wsp* gene sequence to check for *w*Cer3 reads. The library of the Ccap10.3 line only had reads mapping to the *w*Cer4-specific *wsp* and MLST gene sequences, in line with the expectation based on PCR-based analysis [42]. Mapping of read sequences of the RcerHB library to the *w*Cer *wsp* and MLST sequences showed that *w*Cer1 was present as a single infection with no background reads for any other strain. RcerIZ mapped only to *w*Cer1 (moderate) and *w*Cer5 (low); and RcerAS mapped to *w*Cer1 (moderate), *w*Cer2 (high) and *w*Cer5 (low) as stated in Morrow, et al. [24]. Therefore, Ccap10.3 was used to assemble *w*Cer4, and RcerHB was used to assemble *w*Cer1. By using parameters that filtered and excluded *w*Cer1 and *w*Cer2 reads, RcerAS alone was used to assemble *w*Cer5, because mapped reads of RcerIZ to the *w*Cer5 contigs produced low coverage.

To generate the draft genomes of the supergroup A strains *w*Cer1 and *w*Cer4 found as single infections in the sequenced individuals, trimmed paired reads were de novo assembled into contigs using default parameters in CLC Genomics Workbench. Each set of contigs was queried using the complete *Wolbachia* genomes *w*Mel (GenBank: AE017196), *w*Ri (GenBank: CP001391) and *w*Ha (GenBank: NC021089). Those contigs identified as *Wolbachia* sequence were extracted and aligned against *w*Mel using Mauve [70]. The reordered contigs were manually scaffolded and GapFiller [71] extended the sequence and closed the gaps where possible. The *w*Cer1 and *w*Cer4 scaffolds were subsequently realigned with other reference sequences (*w*Ri and *w*Ha), and Gapfilling and mapping were repeated. The scaffolds were finally mapped at 99% similarity over 95% of the read length to verify the genome sequence.

For the supergroup B strain *w*Cer5, assembled contigs from RcerAS were queried by the *w*Pip (GenBank NC_010981) genome. Only the contigs larger than 500 bp and with a match of above 95% were kept, to exclude contigs representing *w*Cer1 or *w*Cer2 in that individual. Contigs were then arranged in order of the *w*Pip genome using Mauve, and reads were mapped at a high stringency of 98% similarity and 90% length to again favour *w*Cer5 reads. GapFiller could not be used because the highest proportion of reads in the RcerAS libraries was for *w*Cer2 and *w*Cer1, respectively, which were at times preferentially incorporated instead of *w*Cer5 sequences, and, therefore, introduced too many errors. RcerAS reads were mapped to *w*Pip at a stringency of 97% for 60–90% of the read length to also extend the *w*Cer5 contig sequences. Any contigs at this stage that had excessively high relative coverage were identified, checked against the *w*Cer1 and *w*Cer2 genomes and removed if identical to *w*Cer1 or *w*Cer2. When polymorphic sequences were found, the alternatives were checked against the known assemblies of *w*Cer1 and *w*Cer2 and removed. This careful approach ensured that the *w*Cer5 draft genome did not include sequences from *w*Cer1 or *w*Cer2. However, it is possible that the assembled *w*Cer5 genome is missing some sequences that were removed, particularly if a region is identical or very similar to *w*Cer1 and *w*Cer2 sequences.

The final draft genomes were mapped at a stringency of 99% over 95% of the read length: reads from RcerHB were mapped to *w*Cer1 (16 contigs); reads from Ccap10.3 were mapped to *w*Cer4 (65 contigs) and reads from RcerAS were mapped to *w*Cer5 (57 contigs).

### Annotation and analysis

Each of the draft genomes *w*Cer1 (RcerHB), *w*Cer4 (Ccap10.3) and *w*Cer5 (RcerAS) were submitted to NCBI. The three genomes plus 16 reference genomes were all annotated using PROKKA v1.13.3 [72] to standardise the subsequent analyses. The completeness of the new genomes was ascertained by comparison to other complete *Wolbachia* genomes via the BUSCO v3.0.2 pipeline for Proteobacteria, which determines the presence of a standardised set of 221 single copy genes in each genome [73]. Prophage regions were annotated using the PHASTER server [74].

OrthoFinder version 2.3.1 [75] was applied with default parameters to the coding sequences identified in *w*Cer1, *w*Cer4 and *w*Cer5 and an additional 16 reference genomes, listed in Table 2. Orthologous genes from these 19 genomes were clustered into orthogroups, and these groupings were visualised using the UpSetR package [76], and also supported multigene phylogenetic analysis and the identification of target gene orthologues, such as *cifA* and *cifB* orthologues.

A subset of coding sequences common to all 19 genomes was aligned and maximum likelihood phylogenetic trees were computed. The set of single copy orthologues identified in OrthoFinder were further filtered for recombinant loci as previously described [24] using PhiPack [77]. Gene (codon) alignments, and subsequent determination of monophyletic adherence to supergroup A and B classifications, were performed in R using the ape package [78]. Maximum likelihood trees were estimated using IQ-TREE [79] from concatenated gene alignments using FASconCAT [80] and a general time reversible base substitution model (GTR + F + R2) as selected by ModelFinder [81].

Orthologues of *cifA* and *cifB* genes were found by locating orthogroups containing the *cifA* and *cifB* genes from *w*Mel [T1], *w*No [T3], *w*Pip [T4] and *w*Stri [T5]. The nomenclature of *cif* gene pairs has recently been proposed to follow the format of *cif*_*wStrain[T1]*_ as an example for a Type I pair [82]. Protein domains within CifA and CifB were identified by HHPred, using databases SCOPe70_2.07, Pfam-A_v34, COG_KOG_v1.0 and SMART_v6.0 [83], furin cleavage sites were detected using PiTou [84] and the gene structures of CifA and CifB were prepared using IBS data visualisation [85]. The gene sequences were codon-aligned in MEGA v7 using Muscle with special consideration of the domains highlighted by HHPred, in Lindsey et al. [23] and the mutagenesis study by Shropshire et al. [54]. The *cifA* alignment excluded orthologues if they were truncated and did not contain the unannotated N-terminal region, or the catalase-rel or DUF domains, because mutations in any of these essential regions can diminish CI and rescue [54]. The *cifB* gene alignment included orthologues only if they contained the unannotated N-terminal region and the two PDDEXK domains common to Type I and Type IV *cifB* genes which have both been determined experimentally to induce CI. Maximum likelihood trees were estimated from the gene alignments as described above, using models TPM3 + F + G4 for *cifA* and TPM3 + F + I + G4 for *cifB*.

*wmk* orthologues were identified in the *w*Cer genomes from the orthogroup containing *w*Mel WD0626 (*wmk*). No orthologues were found in *w*Cer1, but each orthologue from *w*Mel (seven genes), *w*Rec (one gene), *w*Cer2 (eight genes), *w*Cer4 (five genes) and *w*Cer5 (three genes) were codon-aligned in MEGA v7 using Muscle, and the amino acid pairwise distances were calculated.

### Variant analysis of *w*Cer1 and *w*Cer5 genomes in different hosts

Polymorphisms between *w*Cer1 variants of three populations (RcerAS, RcerHB, RcerIZ), and between *w*Cer5 variants of two populations (RcerAS, RcerIZ), were identified by read mapping using CLC Genomics Workbench to the final draft genomes at a similarity of 97% over 97% of the read length, and only properly paired reads were kept. These parameters differed from the parameters used to verify the draft genome sequences because here we wanted to capture strain variation within an individual. For the RcerAS library (comprising *w*Cer1, *w*Cer2 and *w*Cer5), reads were competitively mapped to the *w*Cer2 (GenBank Accession No: SOZK01000000) as well as the *w*Cer1 and *w*Cer5 genomes simultaneously to restrict errors, primarily due to *w*Cer2 reads mapping to the *w*Cer1 genome. This problem was likely to occur because *w*Cer1 and *w*Cer2 are both supergroup A strains and *w*Cer2 reads were more abundant than *w*Cer1 reads and would therefore inflate the outcome of variant detection. The RcerIZ library was competitively mapped to *w*Cer1 and *w*Cer5, and RcerHB was only mapped to *w*Cer1. These stringency parameters allowed for polymorphisms to be detected, while minimising off target reads.

Read mapping was used to (i) generate a library-specific consensus sequence for each strain and (ii) detect variation within individuals for each strain. For *w*Cer1 variant SNP calling, the RcerAS and RcerHB libraries were subsampled (26 million and 10 million reads respectively) to normalize the number of *w*Cer1 reads against the full RcerIZ library that had 258,194 properly paired reads mapped to *w*Cer1 (average 20x coverage). For *w*Cer5 variant SNP calling, the RcerAS library was not subsampled to the level of RcerIZ, because the low number of reads from RcerIZ (51,402 reads) gave very low and sparse coverage (average 4x). The full RcerAS library was used and provided ~18x coverage. In order to aid analysis, variants of *w*Cer2 from two *D. simulans* lines and one *C. capitata* line carrying single infections of *w*Cer2 [24] were also analysed along with *w*Cer2 from RcerAS subsampled to 87 million reads. To determine the consensus sequences, no minimum read number threshold was applied and the majority (> 50% reads) nucleotides were extracted for each *Wolbachia* strain derived from each library to determine a library specific genome. Alignment of these consensus sequences (Additional Files 7, 8 and 9) were used to draw variant genome networks for *w*Cer1, *w*Cer2 and *w*Cer5 using Popart [86]. For variant detection within an individual, the threshold for read coverage was set at five reads, if fewer reads mapped the reference nucleotide was called as default. Variant detection was performed in CLC Genomics Workbench, with variant sites only listed (in Additional Files 10, 11 and 12) for SNPs present at a minimum of 35% of reads. These parameters were selected to highlight prevalent variation and minimise false positives due to sequence errors.

### Mitochondrial genomes

The mitochondrial genome contigs were extracted from each *R. cerasi* de novo assembly via BLASTn match to the *C. capitata* complete mitochondrial genome (GenBank Acc: AJ242872). The sequencing reads were mapped at high stringency (97% similarity and 90% length) and the circular genomes closed and verified by mapping at 99% similarity and 95% length. Protein coding genes (PCGs), tRNAs and rRNAs were annotated using Mitos2 [87] and manually adjusted in line with published annotations of other tephritid mitogenomes. Sequences were aligned in MEGA v7 using MUSCLE, and differences were noted.

SNPs across the three *R. cerasi* mitochondrial genomes were identified by mapping a subsample of each library to the RcerHB mitochondrial genome at 97% similarity and 97% length. Each library was subsampled to achieve approximately 500-fold coverage of the mitogenome, hence RcerHB (530,000 paired reads), RcerIZ (12,000,000 paired reads) and RcerAS (270,000 paired reads) were sampled and mapped to the RcerHB mitogenome. Variant SNPs were called with a low frequency cut-off of 1%, and differences between populations were identified when found at > 99% frequency.

### Linkage of mitochondrial haplotypes and *Wolbachia* strains

A ddRADseq dataset representing 192 *R. cerasi* individuals from six countries (Austria, Germany, Italy, Norway, Portugal and Iran) published as part of a population study [53] was downloaded from the NCBI SRA (Acc. No. SRX6787773). The 273,988,021 raw reads included the barcodes and the modified restriction site at the 5′ end of the sequence. These reads were competitively mapped to the four *Wolbachia* genomes (*w*Cer1, *w*Cer2, *w*Cer4, *w*Cer5) and the mitochondrial genome of RcerHB at 85% of read length and 98% similarity, and only reads specific to a single genome were retained. This low length stringency was chosen so the overhanging barcode of 8 to 10 nucleotides met the parameters. The barcodes were used to identify the samples that mapped to regions on the mitochondrial genome that showed variability. These samples were scored for *Wolbachia* strain presence by examining mapping coverage over the four *Wolbachia* genomes. The threshold selected was at least one perfect read over at least five mapped regions of the genome. This threshold meant that low titre strains were reliably detected (even at one-fold coverage over many regions) but eliminated misallocation of reads to a different *Wolbachia* strain where the genome was incomplete; this could have occurred if *w*Cer5 was present but the conservative approach to its genome assembly resulted in some of its reads mapping to another genome.

Mitochondrial SNPs for each of 46 samples were identified from the ddRadseq mapped reads, tabulated and converted to a genind object and a Euclidean distance matrix using adegenet [88, 89] in R [90]. Samples were grouped as HT1 or HT2, based on presence or absence of *w*Cer2, if they had *w*Cer4 or *w*Cer5, and by country of origin (with Sicily divided into Sicily West and Sicily East). Adonis of the R package vegan [91] was implemented to perform a PERMANOVA to detect differences between the groups.

## Supplementary Information

**Additional file 1.** UpSet graph showing shared *Wolbachia* orthogroups.

**Additional file 2.** PHASTER identification of prophage regions in *w*Cer1, *w*Cer4 and *w*Cer5.

**Additional file 3. ***cifA* and *cifB* locus names from *Wolbachia* strains present in phylogeny.

**Additional file 4.** Mitochondrial genomes and the polymorphic sites between RcerHB (HT1a), RcerIZ (HT1b) and RcerAS (HT2).

**Additional file 5.** Polymorphisms in mitogenomes of three complete mitogenomes of RcerHB (HT1a), RcerIZ (HT1b) and RcerAS (HT2) and an additional 46 samples with SNP representation at 12 of these poymorphic sites from ddRadSeq data. Sample names and location are from Bakovic et al [53], together with associated data.

**Additional file 6.** PERMANOVA of mitochondrial genetic distances between samples grouped by presence of *Wolbachia* strains.

**Additional file 7.** FASTA aligned *w*Cer1 consensus sequences from RcerHB, RcerIZ and RcerAS.

**Additional file 8.** FASTA aligned *w*Cer5 consensus sequences from RcerIZ and RcerAS.

**Additional file 9.** FASTA aligned *w*Cer2 consensus sequences from RcerAS, DsimRC45, DsimRC50 and Ccap88.6.

**Additional file 10.***w*Cer1 variant calling. Reference position refers to the *w*Cer1 genome position after the 16 contigs were joined in order. Reads from three libraries: RcerHB (sampled to 10 million reads), RcerIZ (all reads) and RcerAS (sampled to 26 milion reads), were mapped at 97% length and 97% similarity, and RcerIZ and RcerAS were mapped competitively to the genomes of *w*Cer1 and *w*Cer5; and *w*Cer1, *w*Cer2 and *w*Cer5, respectively. Variants were called with a minimum cut-off of 35% frequency, so a frequency of 65% for a variant is considered homozygous. Location of SNPs within genes is based on PROKKA annotation, and determination of copy number (single or multiple copy) was based on Orthofinder assessment of orthogroups including *w*Cer1, *w*Au and *w*Mel genomes.

**Additional file 11. ***w*Cer5 variant calling. Reference position refers to the *w*Cer5 genome position after the 57 contigs were joined in order. Reads from two libraries: RcerIZ (all reads) and RcerAS (all reads), were mapped at 97% length and 97% similarity, and RcerIZ and RcerAS were mapped competitively to the genomes of *w*Cer1 and *w*Cer5, and *w*Cer1, *w*Cer2 and *w*Cer5, respectively. Variants were called with a minimum cut-off of 35% so a frequency of 65% for a variant is considered homozygous. Location of SNPs within genes is based on PROKKA annotation, and determination of copy number (single or multiple copy) was based on Orthofinder assessment of orthogroups including *w*Cer5, *w*Meg and *w*Pip genomes.

**Additional file 12. ***w*Cer2 variant calling. Reference position refers to the *w*Cer2 genome position after the 11 contigs were joined in order. RcerAS (sampled to 87 million reads) was mapped competitively to the genomes of *w*Cer1, *w*Cer2 and *w*Cer5, respectively at 97% length and 97% similarity. Variants were called with a minimum cut-off of 35% frequency.

## Data Availability

The *w*Cer1, *w*Cer4 and *w*Cer5 genomes were submitted as a Whole Genome Shotgun project (BioProject No. PRJNA668868) at DDBJ/ENA/GenBank under the accessions JADCNC000000000 (*w*Cer1) (https://www.ncbi.nlm.nih.gov/nuccore/JADCNC000000000); JADCND000000000 (*w*Cer4) (https://www.ncbi.nlm.nih.gov/nuccore/JADCND000000000); and JADCNE0000000000 (*w*Cer5) (https://www.ncbi.nlm.nih.gov/nuccore/JADCNE000000000). The versions described in this paper are JADCNC010000000, JADCND010000000 and JADCNE0100000000. Raw reads were submitted to NCBI SRA (sequence read archive), also under BioProject No. PRJNA668868 (https://www.ncbi.nlm.nih.gov/sra/PRJNA668868).
