# Supplementary material for: Genome analyses of four Wolbachia strains and associated mitochondria of Rhagoletis cerasi expose cumulative modularity of cytoplasmic incompatibility factors and cytoplasmic hitchhiking across host populations
Source: BMC Genomics. 2021 Aug 13;22:616. doi: 10.1186/s12864-021-07906-6 (PMC8361831; doi:10.1186/s12864-021-07906-6)
Supplement: Supplementary file 1 — Additional file 1. UpSet graph showing shared Wolbachia orthogroups. [file 12864_2021_7906_MOESM1_ESM.pdf]

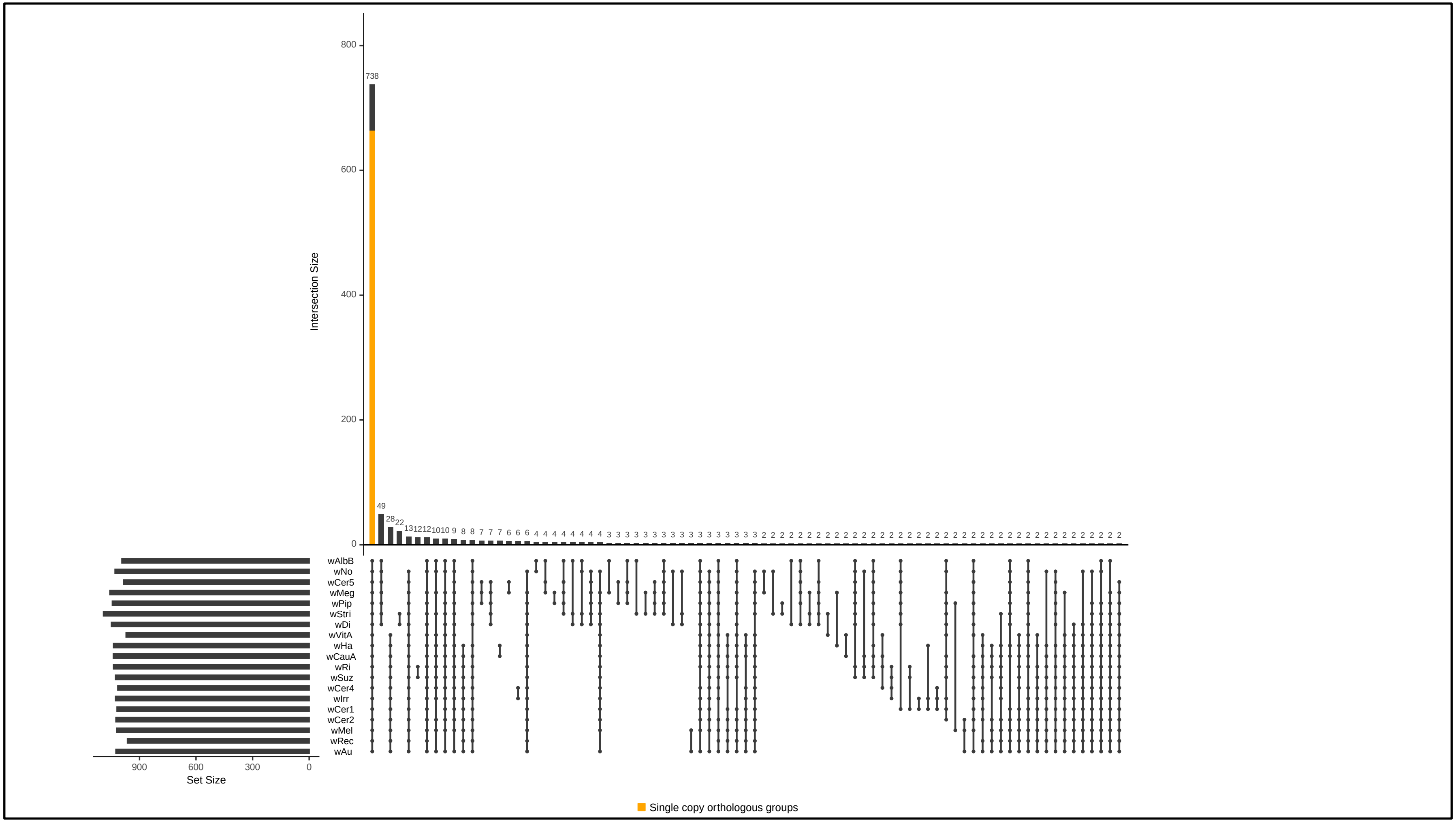

Additional File 1. UpSet graph represents the number of orthogroups that are shared by more than one *Wolbachia* strain as vertical bars, with the subset of single copy orthogroups coloured orange. Nineteen *Wolbachia* strains are listed, with black dots joined by vertical lines denoting membership of the shared group (intersection). Each vertical bar shows the intersection size with the number of orthogroups above each bar. The graph is generated from 1,373 orthogroups, but the minimum intersection size displayed was two strains. Set size is the number of orthogroups represented by each *Wolbachia* strain. The single copy orthologues comprise the bulk of the genes present in all 19 genomes (664 genes) with an additional 74 orthogroups present in all 19 genomes in multiple copies for at least one genome. The largest differentiation of groups was the split between supergroup A and B, with 49 orthologues exclusively and completely in supergroup B (*w*AlbB to *w*Di), and 28 orthologues exclusively and completely in supergroup A (*w*VitA to *w*Au).
